# *In ovo* effect of Soursop (*Annona muricata* L.) leaf extract on hatching and post-hatch performance of Noiler chickens

**DOI:** 10.1016/j.vas.2023.100311

**Published:** 2023-08-22

**Authors:** Timothy T. Kuka, Batomayena Bakoma, Francisca C. Kuka, Benjamin Adjei-Mensah

**Affiliations:** a Regional Center of Excellence in Poultry Science, University of Lome, BSP 1515, Lome, Togo; b Pharmaceutical Sciences Research Laboratory, University of Lome, BSP 1515, Lome, Togo

**Keywords:** Chick mortality, Chick quality, In ovo, Noiler, Soursop, Leaf extract

## Abstract

Pharmacological and nutritional benefits of plant leaf extracts can be harnessed to address the problem of poor chick quality, performance and high mortality, which affect both hatchery and farm managers. This study sought to evaluate the *in ovo* effect of Soursop leaf extract (SLE) on the hatching and post-hatch performance of Noiler chicks. A total of 640 fertile eggs were randomly distributed into four groups of five replicates: (0.25 µg SLE), (0.5 µg SLE), (0.75 µg SLE) and a non-injected control. On day 18 of incubation, 0.2 ml of SLE was injected into the airspace of the eggs. At hatch, embryonic mortality, hatchability, and chick quality were evaluated. Hatched chicks were reared to assess feed intake, weight gain, feed conversion ratio (FCR) and mortality. Results showed no significant difference (*p* > 0.05) in embryonic mortality, hatchability and weight gain among the treatment groups. Chick weight and feed intake were significantly higher (*p* < 0.05) in SLE groups, while FCR and mortality were significantly lower (*p* < 0.05) in SLE groups. It was concluded that *in ovo* injection of SLE improved chick weights, feed conversion and livability of Noiler chicks.

## 1 Introduction

Poultry plays a crucial role in food security and nutrition. The increasing demand for poultry is expected to continue due to human population growth, nutritional/health awareness, income levels and urbanization (Miller et al., 2022). This calls for increased poultry production, of which day-old chicks are the primary input. However, the poultry industry has over time battled with disease control, efficient production, product quality, and reasonable production costs (Hafez & Attia, 2020). Apart from the recent surge in feedstuff prices and other inputs which posed a difficulty for many producers, high chick mortality and poor flock performance are serious challenges affecting efficient poultry production which must be adequately tackled to maximize profit and meet the expected demand for poultry products (Yassin et al., 2009).

Concerted efforts made in the management of chick mortality and improved flock performance are more dependent on the use of synthetic antibiotics for the prevention of infections and growth-boosting, which have been criticized and banned in food animals (Ezzat Abd El-Hack et al., 2016; Lodang et al., 2020). More so, these approaches are post-hatch based whereas the issues of chick mortality and poor performance are strongly associated with the quality of day-old chicks which is in turn affected by incubation and pre-incubation conditions (Decuypere & Bruggeman, 2007). The quality of day-old chicks is important to both hatchery and farm managers. High-quality day-old chicks enable commercial hatcheries to maximize sales and profit, while commercial farms prefer high-quality chicks as key determinants of a successful and profitable production cycle (Narinc, 2022).

However, obtaining a large number of quality chicks, which can express their full genetic potential, under favourable rearing conditions is only possible with adequate improvement of egg incubation technology and hatching results (Nowaczewski et al., 2022). Improving hatchability, chick quality, and performance will mean additional profit for hatchery and farm managers.

*In ovo* technology, which is used commercially for embryonic vaccination (Araujo et al., 2019), is a viable tool for developing a wholesome chick quality and performance solution. Researchers have adopted this technology for the delivery of nutrients, (Groff-Urayama et al., 2019; Hajati et al., 2014), antibiotics (Hadi et al., 2014) and other supportive substances including plant extracts to avian embryos during incubation (Coşkun et al., 2014a, b; N'nanle et al., 2017; Sogunle et al., 2022; Oke et al., 2021), to improve hatching and growth performance of chickens as well as preventing transmission of diseases from eggs to chicks. Though the results have been promising, there exist some contradictions mainly due to the varying nutrient compositions of the substances, extraction techniques employed and the rate of injection (N'nanle et al., 2017; Sogunle et al., 2022; Oke et al., 2021), which necessitate the exploration of more materials, especially plant materials to validate the *in ovo* injection approach.

Plants are a rich source of essential nutrients (Samtiya et al., 2021), chemicals and other active components (Kumar et al., 2021), which have been used for health maintenance in humans and performance enhancement in poultry production (Dhama et al., 2015; Gheisar & Kim, 2018; Gheisar et al., 2015, b). *Annona muricata* is a plant with leaves rich in proteins, carbohydrates, beta-carotene, ascorbic acid, flavonoids, alkaloids, saponins, tannins, and cardiac glycosides. The leaves are noted for anti-microbial, anti-oxidant, anti-inflammatory, and anti-parasitic effects (Agu & Okolie, 2017; Usunobun et al., 2015; Usunobun and Okolie, 2015). In vivo administration of *Annona muricata* leaf extract has been reported to prevent diseases, promote intestinal health and growth performance in broiler chickens (Oluwayinka et al., 2017; Kuka, Agedeson, & Ebiaku, 2022).

Although soursop leaf extract positively impacts poultry performance in vivo, its potential effects on chicken embryonic development, hatching and post-hatch performance have not yet been reported. Based on the nutrient and phytochemical profile of soursop leaf extract, we hypothesized that *in ovo* administration of soursop leaf extract will improve incubation and post-hatch performance of chickens. Therefore, this study was designed to investigate the *in ovo* effects of soursop leaf extract on hatchability, embryo mortality, chick quality and post-hatch performance of Noiler chickens. Noiler chicken is a dual-purpose breed of chicken developed by Amo Farm Sieberer Hatchery Limited, Nigeria, for small-holder farmers to address the challenges of food insecurity and financial dependency among the rural populace, especially women. Noiler is a breed developed to thrive well on low-quality feeds and provide good-quality meat and eggs. The roosters are targeted mainly for meat and mature between 3–6 months while the hens are mostly bred for egg-laying purposes, and begin laying between 5–6 months (Livestocking, 2019).

## 2 Materials and methods

The present study was conducted at the hatchery and experimental units of the Regional Center of Excellence in Poultry Science (CERSA), following the guidelines of the Canadian Council on Animal Care (2009), under the approval (008/2021/BC-BPA/FDS-UL) and supervision of the research and development team leaders, University of Lome, Togo.

### 2.1 Preparation of the leaf extract

Soursop leaves were harvested from the suburb of Lome, Togo. The leaves were air-dried under air condition (20 °C) and milled into powder for use. The extraction was done following a slight modification of the procedure of Cheng et al. (2021). Five hundred (500) g of soursop leaf powder were macerated in 5.5 litres of ethanol (80%). The mixture was shaken for 72 h, after which it was first filtered through cotton wool in a filter funnel and finally through a coffee filter (# 6). The filtrate was concentrated using a rotary evaporator with a chiller and vacuum pump at 40 °C to obtain a sticky leaf extract and preserved under fridge conditions for reconstitution and use. Ten (10) mg of the extract were reconstituted with 10 mL of saline solution (0.9%) to obtain the stock solution (1000 µg). One ml of the stock solution was again reconstituted with 100 ml of saline solution to produce a 10 µg working solution. Finally, 2.5 ml, 5.0 ml and 7.5 ml of the working solution were reconstituted with 10 ml of saline solution, respectively, to obtain 0.25, 0.5 and 0.75 µg concentrations used for the injection.

### 2.2 Experimental design and injection

A completely randomized design was used for the experiment. Seven hundred and fifty-six (756) hatchable eggs of a dual-purpose (Noiler) chicken were obtained based on the hatching performance records from a breeder stock of 38 weeks old in Lome. The eggs were weighed individually and incubated at a temperature of 37.7 °C and relative humidity of 60% with automatic turning at an hour interval in a Royal Pas Reform (SmartPro ^TM^) combi incubator, Netherlands. On the 18th day of incubation, eggs were candled to remove infertile ones and eggs with evidence of livinging embryos were randomly assigned to four groups of 160 eggs each. Groups one, two and three were injected with the following concentrations of soursop leaf extract 0.25 µg, 0.5 µg and 0.75 µg; the eggs were returned to the incubator and allowed for six hours before transferring them to the hatching baskets. In the hatcher, the temperature was maintained but relative humidity increased to 70% and the eggs were observed for hatching according to their groups. Before injection, two holes were made on the broad side of the eggs using 21 g needles to ease delivery of the extract and avoid tension in the eggs during delivery of the extract, the extract was injected using an automatic syringe, and thereafter, the holes were sealed with a adhesive tape. No group of eggs was injected with saline solution because previous studies (Atan & Kop-Bozbay, 2021; Karamik & Kop-Bozbay, 2020), reported no significant difference between the saline-injected and non-injected eggs. All the injected eggs received 0.2 ml of the extract in the air space.

### 2.3 Data and sample collection

The percentage hatchability was calculated by this formula:

$$Hatchability\;\left(\%\right)=\frac{Number\;of\;hatched\;chicks}{Number\;of\;fertile\;eggs}\times 100$$

Embryonic mortality was obtained by breaking the unhatched eggs to confirm dead embryo or rotten eggs, and the stage of mortality, embryo mortality was calculated as follows:

$$Mortality\;\left(\%\right)=\frac{Number\;of\;dead\;chicks}{Number\;of\;fertile\;eggs}\times 100$$

Fifty (50) of the hatched chicks (10 chicks per replicate) from each group were weighed and assessed for chick quality using the Tona score: activity, the eyes, navel, legs, beak, retracted yolk, remaining membrane, remaining yolk down and appearance (Tona et al., 2003). Each group of the hatched chicks were randomly divided into five (5) replicates of 20 chicks and reared for 12 weeks to observe the effect of *in ovo* extract on the post-hatch performance of the chicks. The birds had 12 h of light and unlimited access to feed and water. They were reared in open-sided poultry pens with a stocking density of 10 birds per square meter on a floor covered with 4 cm thick wood shavings. Prophylaxes and bird vaccinations were given out in accordance with Noiler breed guidelines. The birds were fed a starter diet containing 20% crude protein and 2800 Kcal/kg metabolizable energy from day one to five weeks and crude protein and metabolizable energy of 18% and 2700 Kcal/kg from six to twelve weeks for the finisher diet (Table 1). Feed intake, weight gain, feed conversion ratio and mortality were recorded and calculated at week one and week 12. Two birds per replicate were starved of feed but not water for 12 h and slaughtered. The carcass was cut into parts and weighed using a sensitive scale. After carefully removing the heart, gizzard and liver, the weight of each replicate was calculated as a percentage of the live weight.

$$\;Averagedailyweightgain\;=\frac{\;Weeklyweightgain\;(g)}{7}$$

$$\;Feedconversionratio\;=\frac{\;Feedintake\;(g)}{\;Weightgain\;(g)}$$

$$Dressed\;\left(\%\right)=\frac{Dressed\;weight\;\left(g\right)}{Live\;wieght\;\left(g\right)}\;x\;100$$

$$Cut-up\;parts\;\left(\%\right)=\frac{Weight\;of\;cut-up\;part}{Dressed\;weight}x\;100$$

$$Relative\;organ\;weight\;\left(\%\right)=\frac{Organ\;weight\;\left(g\right)}{Live\;weight\;\left(g\right)}\;x\;100$$

**Table 1:** Composition of the experimental diets for starter and finisher Noiler chickens.

Ingredients(kg)	Starter diet	Finisher diet
Maize	53.00	54.00
Soya bean meal (46%)	33.00	25.00
Wheat bran	7.00	14.00
Concentrate (5% JLC)*	5.00	5.00
Oyster shells	2.00	2.00
Total	100.00	100.00
*Nutrients composition*		
Crude protein (%)	20.00	18.00
Metabolizable Energy (kcal)	2800.00	2700.00
Crude fiber (%)	4.01	6.20
Calcium (%)	1.04	1.02
Phosphorus (%)	0.45	0.45
Methionine (%)	0.50	0.50
Lysine (%)	1.00	1.00

⁎ Jubaili Layer Concentrate: crude protein, 30%; crude fats, 2%, calcium, 4%; phosporus, 4.1%; Lysine, 2.0%; methionine, 3.0%; methionine + cystine, 3.5%; sodium, 2.0%, Met-energy, 2280 Kcal/kg; vitamins; minerals, enzymes.

### 2.4 Statistical Analysis

The entire data collected was computed using percentages and one-way analysis of variance (ANOVA) in a completely randomized design using the procedure of SPSS Software (Version 23). Differences between treatment means were compared using Tukey's honestly significant difference test at *p* ≤ 0.05. The following model was used: Yij = µ + Ti + Eij; where µ was the population mean; Ti, was the effect of each *in ovo* injection treatment and Eij, was the residual error.

## 3 Results and discussion

### 3.1 Hatch performance

The results of the effect of *in ovo* administration of soursop leaf extract on embryo mortality, hatchability and chicks’ weight are presented in Table 2. No significant effect was observed on the hatchability (*p* = 0.35) and embryonic mortality (*p* = 0.24). Chick weights were significantly higher (*p* = 0.01) in the injected groups than in the non-injected group. Increased chick weight in this study can be attributed to the antioxidant capacity of SLE (Widyastuti & Rahayu, 2017) and the nutrient content of the leaves (Kuka, Agedeson, & Ebiaku, 2022). It could also be said that the injection process as well as the 0.75 µg concentration were not harmful to the embryos.

**Table 2:** *In ovo* effect of soursop leaf extract on hatchability, embryo mortality and chick weight of Noiler chicks.

	Treatments					
Parameters	Control	0.25 µg SLE	0.50 µg SLE	0.75 µg SLE	SEM^1^	P-value^2^
Hatchability of fertile eggs (%)	90.18	89.00	89.36	90.79	0.044	0.350
Rotten eggs (%)	1.00	1.5	1.52	1.00	0.032	0.323
Live but unhatched (%)	0.85	1.25	1.02	0.32	0.046	0.420
Embryo Mortality (%)	7.97	8.25	8.10	7.97	0.051	0.244
Chick Weight (g)	36.051^b^	37.81^a^	37.52^a^	37.67^a^	0.11	0.014

Abbreviation: SLE- Soursop leaf extract **^a,b^**Means with different superscripts are significantly different.

1 SEM = pooled standard error of means.

2 P-value = Probability

It is established that later stage development of the embryo and mobilization for hatching usually requires high energy input. Unfortunately, the energy reserve of the egg is not sufficient for these activities; hence the embryo resorts to energy production through gluconeogenesis which degrades proteins from the muscles leading to loss of weight by the chick, poor hatching and even death (Asaa et al., 2022; Coskun, Guray, Ahmet and Aylin, 2014b). In another sense, high metabolic activities at the late-term incubation induce oxidative stress for the embryo leading to reduced performance and survival of the chick (Asaa et al., 2022). Nevertheless, i*n ovo* injection of nutrients, antioxidants and substances containing either or both of them has been proven to reverse the notable effects (Asaa et al., 2022; Das et al., 2021; Zhai et al., 2011).

The result of the present study corresponds to other findings on *in ovo* plant extracts but with some degree of variations. Coşkun et al. (2014a), reported a similarity in hatchability and increased hatch weight of chicks with *in ovo* administration of pollen extract. Similarly, Aygun (2016), observed that *in ovo* administration of 1% propolis extract did not significantly affect hatchability as well as embryo mortality of broiler chicken but higher levels decreased hatchability, this was also observed by Heidary et al., and Mohebalian (2020). On the other hand, Kop-Bozbay and Göneci (2023), reported that cinnamon and ginger groups had higher hatchability and chick quality, while N'nanle et al. (2017), reported increased hatchability and chick weight at lower concentrations *of in ovo* moringa leaf extract but reduced hatchability at higher concentrations.

The finding of this current study is consistent with the report of El-Kholy et al., and El-Said (2021) who administered *in ovo* cinnamon, thyme and clove extracts, and Akosile et al. (2023) who injected clove and cinnamon extract in broiler chicken eggs. Both researchers reported increased chick weight while Ngueda et al. (2021), who injected 0.5 µg of cassava leaf extract in broiler chicken eggs reported similarity in hatchability and mortality rate.

Embryo mortality is a crucial determinant of high or low hatchability, high embryo mortality will result in low hatchability and vice versa. A careful consideration of these studies reveals that plant extracts could be harmful or beneficial to the developing embryo at certain concentrations, thereby affecting hatchability. Furthermore, the studies all revealed that the *administration* of plant extracts increased chick weight. El-Kholy et al. (2021) suggested that this increase is due to the improved antioxidant status of embryos derived from the extract which alleviates hatch-related oxidative stress and protects skeletal muscle stem cells from oxidative damage.

### 3.2 Chick quality

Table 3 shows the effect of *in ovo* administration of soursop leaf extract on chick quality of Noiler chickens. Significant differences were observed in down and appearance, remaining membrane and overall quality scores among the groups. It was observed that overall chick quality scores decreased (*p* = 0.01) with lower levels of soursop extract but increased and became similar with the non-injected control group at 0.75 µg of SLE. This suggests that tempering with incubated eggs in terms of perforation without sufficient supplementation may significantly affect the health status and quality score of chick, as the same trend was observed in embryo mortality. The effect of SLE on chick quality scores was also observed to be dependent on the concentration of the extract. Soursop leaves are rich in nutrients and phytochemicals such as β-carotenes, ascorbic acid, flavonoids, saponins, alkaloids, tannins and glycosides (Agu & Okolie, 2017). These may have exhibited a free-radical-scavenging ability to reduce oxidative stress and improve the embryo's nutrition.

**Table 3:** *In ovo* effect of soursop leaf extract on chick quality of day-old Noiler chicks.

	Treatments					
Parameters	Control	0.25 µg SLE	0.50 µg SLE	0.75 µg SLE	SEM^1^	P-value^2^
Activity	6.00	6.00	6.00	6.00	0.000	-
Down & appearance	9.92^ab^	9.64^b^	9.76^ab^	9.96^a^	0.041	0.018
Retracted yolk	12.00	11.96	12.00	12.00	0.010	0.394
Eyes	16.00	16.00	15.84	16.00	0.040	0.394
Legs	16.00	15.20	15.04	16.00	0.152	0.035
Naval area	11.52	11.64	11.76	11.64	0.101	0.874
Remaining membrane	11.76^a^	11.04^b^	11.84^a^	12.00^a^	0.079	0.000
Remaining yolk	15.04	14.16	14.48	14.64	0.144	0.185
Total quality score	98.24^a^	95.64^b^	96.72^ab^	98.24^a^	0.329	0.010

Abbreviation: SLE- Soursop leaf extract **^a,b^** Means with different superscripts are significantly different.

1 SEM = pooled standard error of means.

2 P-value = Probability.

The result of this study agrees with Ngueda et al. (2021), who reported that *in ovo* cassava leaf extract caused a significant increase in the overall quality scores of Sasso broiler chicks. On the contrary, N'nanle et al. (2017) did not observe any differences when moringa leaf extract was administered. There is not enough information on the leaf extract's effect on chickens’ chick quality for further comparisons.

### 3.3 Post-hatch performance

The result of the *in ovo* effect of soursop leaf extract on the post-hatch performance of Noiler chicks is shown in Tables 4 and 5. Significant differences were observed in feed consumption (*P* < 0.0001), feed conversion (*P* = 0.03) and percentage mortality (*P* = 0.04) of Noiler chicks during the first week of rearing. *In ovo* SLE increased feed consumption of the chicks with a significantly higher (*p* < 0.0001) value in the 0.25 µg group compared to the control and other groups. Nevertheless, this did not improve weight gain (*p* = 0.38). No significant difference was observed in all the parameters at the finisher phase. The feed conversion ratio improved with a higher concentration (0.75 µg) of *in ovo* SLE in Noiler chicks during the first-week post-hatch. Aromatic plants and their extracts can favourably stimulate endogenous digestive secretions and establish intestinal epithelial structures to influence gut functions and feed efficiency (Jang et al., 2007; Yang et al., 2019). Improved feed conversion in this study can be traced to the anti-inflammatory, gastro-protective, and digestive-enhancing properties of soursop leaves (Gavamukulya et al., 2017).

**Table 4:** *In ovo* effect of soursop leaf extract on growth performance of Noiler chicks at seven days post-hatch.

	Treatments					
Parameter	Control	0.25 µg SLE	0.50 µg SLE	0.75 µg SLE	SEM^1^	P-value^2^
Total weight gain (g)	1964.70	1700.32	1898.08	2110.23	51.703	0.021
Average weight gain (g/d)	7.23	7.46	7.98	7.78	1.090	0.387
Average feed intake (g/d)	10.17 ^c^	11.59 ^a^	11.06 ^b^	10.45 ^bc^	1.259	0.000
Feed conversion ratio	1.41^ab^	1.55^a^	1.39^ab^	1.34^b^	0.033	0.0329
Mortality (%)	5.25^a^	3.75^ab^	2.50^b^	5.00^ab^	0.407	0.041

Abbreviation: SLE- Soursop leaf extract ^a-c^ Means with different superscripts are significantly different.

1 SEM = pooled standard error of means.

2 P-value = Probability.

**Table 5:** *In ovo* effect of soursop leaf extract on growth performance of finisher Noiler chickens.

	Treatment groups					
Parameters	Control	0.25 µg SLE	0.50 µg SLE	0.75 µg SLE	SEM^1^	P-value^2^
Total weight gain (g)	1551.65	1574.59	1583.63	1557.43	24.248	0.972
Final weight (g)	1587.70	1612.40	1621.30	1594.95	24.333	0.968
Average weight gain (g/d)	18.47	18.75	18.85	18.54	0.289	0.972
Average feed intake (g/d)	54.75	59.82	51.10	46.60	1.994	0.100
Feed conversion ratio	2.96	3.21	2.71	2.53	0.105	0.107

Abbreviation: SLE- Soursop leaf extract **^a,b^** Means with different superscripts are significantly different.

1 SEM = pooled standard error of means.

2 P-value = Probability.

Feed conversion ratio is a critical index for evaluating the performance of a flock, it reveals how efficiently the flock can convert the feed consumed into muscles (meat). A low feed conversion ratio means the birds have utilized the feed consumed to put up muscles closely related in weight to the amount of feed. Since the highest cost of production goes to feeding, it is an advantage to the farmer when the feed conversion ratio of the flock is the lowest. This result partly agrees with that of El-Kholy et al. (2021) who reported improved feed conversion with *in ovo* administration of cinnamon, thyme and clove extract without difference in feed intake at both starter and finisher phases of broiler chicken. This result differs from the findings of other researchers who did not observe a difference in feed conversion ratio (Hajati et al., 2014; Ngueda et al., 2021).

*In ovo* administration of SLE significantly reduced (*p* = 0.04) chick mortality at 0.5 µg compared to the un-injected control and the other groups. Mortality rate especially in chicks is a factor that significantly affects the efficiency of production leading to a high loss of resources. A low mortality rate coupled with proper management can improve the return on investment of the farmer. Obviously, *in ovo* SLE has a positive impact on Noiler chicken embryos as reflected in chicks weight, chick quality, feed conversion and post-hatch mortality. These effects can be due to the antioxidant, antibacterial and nutritional properties of soursop leaves (Gavamukulya et al., 2017). In vivo administration of soursop leaf extract has been reported to have improved intestinal morphology, reduced intestinal microbes, and improved feed conversion ratio in broiler chickens (Kuka, Agedeson, & Ebiaku, 2022). Low chick mortality implies a good startup for the farmer which in conjunction with other management practices will give a profitable rearing cycle. The hatchery would as well sustain a reputation among farmers for producing chicks that can live their best.

### 3.4 Carcass characteristics

Table 6 shows the carcass characteristics of finisher Noiler chicken. No significant differences were observed in the dressed percentage, cut parts and organs except for neck and shank cuts. These differences could possibly be as a result of cutting. This result is different from the study of El-Kholy et al. (2021) who recorded significant differences in carcass and organ weights. The lack of difference in carcass characteristics is due to similarities in the final live weight of the chickens.

**Table 6:** *In ovo* effect of soursop leaf extract on carcass characteristics of finisher Noiler chicken.

	Treatment groups					
Parameters	Control	0.25 µg SLE	0.50 µg SLE	0.75 µg SLE	SEM^1^	P-value^2^
Live weight (g)	1604.50	1553.43	1499.88	1565.90	27.398	0.646
Dressed weight (g)	1230.68	1156.70	1117.95	1210.00	23.364	0.331
Dressed %	76.78	74.38	74.50	77.25	0.527	0.090
Breast (%)	23.34	22.55	22.59	23.08	0.185	0.029
Thigh (%)	15.91	14.88	15.08	15.54	0.173	0.041
Drum stick (%)	14.20	13.87	13.94	14.17	0.308	0.000
Wing (%)	12.87	12.28	12.42	12.86	0.158	0.109
Neck (%)	6.26^b^	8.72^a^	8.73^a^	6.91^b^	0.316	0.000
Shanks (%)	8.43^a^	6.85^b^	6.75^b^	8.11^a^	0.288	0.003
Back (%)	19.24	20.21	20.49	19.65	0.409	0.315
Heart (%)	0.56	0.46	0.53	0.48	0.019	0.214
Liver (%)	2.04	1.90	2.10	2.03	0.047	0.538
Gizzard (%)	2.44	2.40	2.67	2.32	0.068	0.326

Abbreviation: SLE- Soursop leaf extract **^a,b^** Means with different superscripts are significantly different.

1 SEM = pooled standard error of means.

2 P-value = Probability.

## 4 Conclusion

*In ovo* supplementation of soursop leaf extract increased the weights of Noiler chicks at hatch, and 0.75 µg of SLE improved chick quality and feed conversion ratio. However, a low concentration of SLE (0.25 µg) negatively affected the chick quality of Noiler. *In ovo* supplementation of SLE also reduced the chick mortality at 0.5 µg. To sum up, *in ovo administration of* Soursop leaf extract improved chick weight, feed efficiency and livability during the juvenile stage of Noiler chicks. We recommend that higher concentrations of soursop leaf extract should be studied as well as different injection sites should be considered to properly study the effect of soursop leaf extract.

## Funding disclosure

This work was funded by the Regional Center of Excellence in Poultry Science (CERSA) under the 10.13039/100004421World Bank Group [IDA 5424].

## Ethical Statement for Solid State Ionics

Hereby, I Timothy Tartenger Kuka consciously assure that for the manuscript/***In ovo* effect of Soursop (*Annona muricata* L.) leaf extract on hatching and post-hatch performance of Noiler chickens,** the following is fulfilled:

1) This material is the authors' own original work, which has not been previously published elsewhere.
2) The paper is not currently being considered for publication elsewhere.
3) The paper reflects the authors' own research and analysis in a truthful and complete manner.
4) The paper properly credits the meaningful contributions of co-authors and co-researchers.
5) The results are appropriately placed in the context of prior and existing research.
6) All sources used are properly disclosed (correct citation). Literally copying of text must be indicated as such by using quotation marks and giving proper reference.
7) All authors have been personally and actively involved in substantial work leading to the paper, and will take public responsibility for its content.

The violation of the Ethical Statement rules may result in severe consequences.

To verify originality, your article may be checked by the originality detection software iThenticate. See also http://www.elsevier.com/editors/plagdetect.

I agree with the above statements and declare that this submission follows the policies of Solid State Ionics as outlined in the Guide for Authors and in the Ethical Statement.

## Declaration of Competing Interest

We the authors (T.T. Kuka, B. Bakoma, F.C. Kuka and B. Adjei-Mensah) of the accompanying article, write to declare that there is no personal or professional conflict of interest with our work.
